# Kinetic inductance driven nanoscale 2D and 3D THz transmission lines

**DOI:** 10.1038/srep25303

**Published:** 2016-05-03

**Authors:** S. Hossein Mousavi, Ian A. D. Williamson, Zheng Wang

**Affiliations:** 1 Microelectronics Research Center, Department of Electrical and Computer Engineering, The University of Texas at Austin, Austin, TX 78758 USA

## Abstract

We examine the unusual dispersion and attenuation of transverse electromagnetic waves in the few-THz regime on nanoscale graphene and copper transmission lines. Conventionally, such propagation has been considered to be highly dispersive, due to the RC time constant-driven voltage diffusion below 1 THz and plasmonic effects at higher optical frequencies. Our numerical modeling across the microwave, THz, and optical frequency ranges reveals that the conductor kinetic inductance creates an ultra-broadband linear-dispersion and constant-attenuation region in the THz regime. This so-called LC region is an ideal characteristic that is known to be absent in macro-scale transmission lines. The kinetic-LC frequency range is dictated by the structural dimensionality and the free-carrier scattering rate of the conductor material. Moreover, up to 40x wavelength reduction is observed in graphene transmission lines.

Transmission lines, one of the simplest forms of single-mode electromagnetic waveguides, are widely used for signaling in digital, analog, and optoelectronic systems. Transmission lines generally consist of two or more parallel conductors that guide a transverse electromagnetic (TEM) wave over many wavelengths^1^,^2^. Ideal TEM waveguides, in which perfect conductors are used, support phase propagation that is not band-limited. In practice however, the finite conductivity of the metal conductors results in phase-propagating TEM modes that have a low frequency cut off (also known as the LC onset frequency) above which, the line’s inductive impedance exceeds its resistance. Crossing this cutoff from low to high frequencies causes the governing telegrapher’s equation to change from an RC time constant-diffusion equation to a wave equation that supports phase propagation. This phase propagation is a crucial property for phase-matching in traveling-wave modulators^3^,^4^. The finite metal conductivity also causes such phase-propagating TEM modes to be band-limited at high frequencies by the skin effect^1^. Practically, the skin effect imparts a square-root frequency dependence on the signal attenuation^5^ and limits the maximum length of board- and chip-level interconnects^6^,^7^, the bandwidth of traveling-wave modulators^8^,^9^,^10^,^11^, and the spatial resolution of time-domain reflectometry systems^12^. In fact, the lateral dimensions of practical transmission lines combined with the finite conductivity causes the LC onset frequency and the skin effect cut off to coincide, resulting in a negligible bandwidth for phase-propagating TEM modes^1^.

Two recent advancements have made it intriguing to examine the dispersion of TEM waves in nanoscale transmission lines: state-of-the-art fabrication processes have allowed the dimension of transmission lines to fall below the skin depth; graphene has emerged as a practical conductor with large kinetic inductance^13^,^14^,^15^,^16^,^17^,^18^. Our investigation reveals that both of these points can be exploited to suppress the skin effect and subsequently realize frequency-independent attenuation. The kinetic inductance stems from the kinetic energy of oscillating free charges, and is significant in superconductors^19^ at microwave frequencies and metals at optical frequencies^20^. Graphene is remarkable in that it has a dominant kinetic inductance at relatively low THz frequencies^21^. This large kinetic inductance has been exploited in infinite sheets^21^,^22^,^23^ and nano-ribbons^24^,^25^,^26^ for ultra-short-wavelength graphene plasmons. Although various graphene transmission line structures have been studied in the microwave^24^,^27^,^28^ and optical regimes^23^,^25^, no comprehensive study of graphene transmission lines has been conducted to map the various line performance regions (RC, LC, skin effect, and plasmonic regimes) over a wide range of graphene sizes and operating frequency ranges.

In this article, we carry out a first-principle numerical and analytical study on the effect of material kinetic inductance on the propagation and attenuation of the transverse electromagnetic modes in nanoscale and microscale transmission lines. We explore the potential of the kinetic inductance in providing a usable broadband LC response for signaling, transducing and sensing applications. Over a vast space, spanning 10^6^–fold variation in dimensions (nm through cm) and 10^4^–fold variation in frequency (10 GHz–100 THz), we examine the conditions for the kinetic inductance to produce a surprisingly broadband LC region for small-scale transmission lines. Investigating both graphene and copper conductors, we develop a map of the performance regions for 2D and 3D materials, contrast the significant differences in their scaling laws, and explain dominant physical mechanisms that limit their performance.

## Results

### Transmission line model

**Figure 1:**
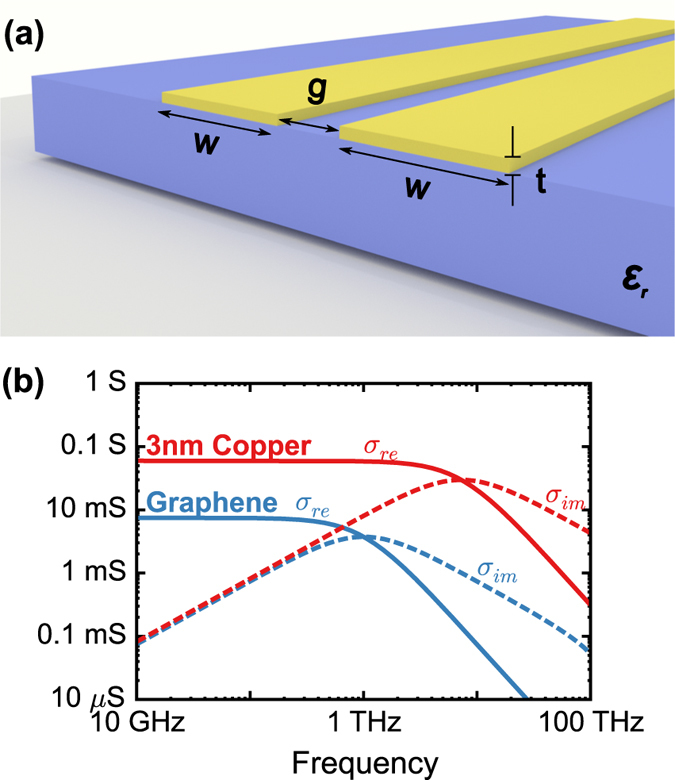
(**a**) Schematic of a coplanar stripline consisting of two parallel conductors on a SiO_2_ substrate (*ε*_*r*_ = 2.25) (**b**) Complex-valued surface conductivity of single-layer graphene (blue) compared with the effective surface conductivity of a 3 nm copper film (red). Graphene has a Fermi level of 0.4 eV and a scattering rate of 1 THz and copper has a scattering rate of 7.26 THz and a DC conductivity of 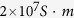.

Among various transmission line structures, we focus on the coplanar stripline (CPS) shown in Fig. 1(a) because it exhibits the typical skin-effect-induced attenuation and it has been widely used in integrated systems^29^ and traveling-wave modulators^10^. The CPS consists of two parallel conductors on top of a dielectric substrate^30^,^31^. We consider two distinct CPS designs with two different conductor materials: one with single-layer graphene ribbons and the second with copper wires. The copper wires have a finite thickness, *t*, and the graphene ribbons are treated as an infinitesimally-thin surface conductor. At THz frequencies and below, both materials are accurately modeled with a Drude conductivity^22^,


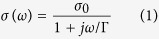


where Γ is the phenomenological scattering rate of the free carriers^21^, and results in the propagation loss of the TEM mode. *σ*_0_ corresponds to the bulk DC conductivity for copper and surface conductivity for graphene. For the purposes of this paper, graphene has been modeled as an infinitesimally-thin conducting surface. Scattering due to surface roughness, finite temperature effects and spatial dispersion are neglected. The effective surface conductivity (*i.e.*, 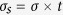) of a 3 nm-thick copper film is roughly 10× larger than the graphene surface conductivity (Fig. 1b). Thicker copper films are used in the rest of this paper, implying an even higher conductance for copper lines. The copper scattering rate is taken to be 7.26 THz^32^ while the graphene scattering rate is 1 THz, which is an experimentally achievable value^33^,^34^. Note that these values are representative and the main points of this paper do not rely on a specific value of Γ or 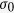.

The underlying physical mechanisms leading to frequency-dependent attenuation in transmission line modes are captured by the circuit parameters in the solution to the telegrapher’s equations. Since we limit our discussion to lines with lateral dimensions that are much smaller than the free-space wavelength, this analytical model is accurate and yields comparable results to ab-initio solutions to Maxwell’s equations^1^,^20^. The real and imaginary parts of the complex-valued propagation constant^2^, 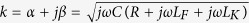, reflect the attenuation (*α*) and spatial phase evolution (*β*), respectively. The circuit parameters *C*, *R*, 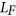, and 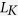 represent the inter-conductor capacitance, conductor resistance, Faraday inductance, and kinetic inductance per unit length, respectively. The LC region occurs at high enough frequencies, where 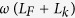 exceeds *R*, but at low enough frequencies where *R*, 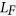, and 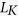 remain constant. Unlike the highly dispersive RC region at lower frequencies, the LC region exhibits linear group velocity and constant attenuation. However, at high frequencies, *R* and 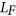 usually vary with frequency due to the non-uniform conduction current distribution. In particular, a large part of 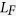
comes from the magnetic energy residing inside the conductors, which can be greatly reduced by current crowding to the conductor surface. As the current redistributes itself, it minimizes the total impedance, 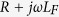. Current crowding occurs quickly above the LC on-set frequency to reduce 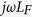, at the cost of increased and frequency-dependent resistance and attenuation^1^. In other words, without the kinetic inductance, the onset frequency of the skin effect and the LC region are very close, because the magnetic flux within the conductors is a major contributor to the total impedance. In order to obtain a large LC bandwidth without kinetic inductance, a large conductor separation would be needed to increase the ratio of the external to internal inductance.

### Kinetic LC response

In contrast to the Faraday inductance, the kinetic inductance stems from a material constant, the complex-valued conductivity. The kinetic inductive impedance is proportional to the imaginary part of the reciprocal of the conductivity, while the resistive impedance relates to the real part. Physically, they contribute to the power stored or dissipated, respectively, in the conduction currents:


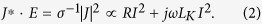


*J* is the surface or volume current density produced by an electric field, *E*. From this equation follows an important relation between the kinetic inductance and the resistance:


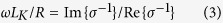


This ratio reduces to a material constant and hence, may be frequency dependent but is completely independent of the geometry of the transmission line. Therefore, similarly to the resistance, the kinetic inductance is also inversely proportional to the current carrying area, *A* (or width *w* in the case of graphene), but unlike the resistance, it is related to the imaginary part of the inverse conductivity, 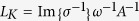, so it becomes significant only at high frequencies.

When the conductivity is represented by a Drude model, Eq. (3) simplifies even further to 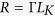. Below the skin effect onset, both *R* and 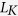 are frequency-independent, even at frequencies above the scattering rate. In such regime, the kinetic inductance is given by 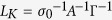. In contrast to the Faraday inductance, the kinetic inductance *increases* with current crowding (i.e., when the conduction area becomes smaller); lines with a dominant kinetic inductance suppress current crowding because current favors a distribution of lowest total impedance.

Nanoscale lines are where the kinetic inductance typically exceeds the Faraday inductance. Generally, the Faraday inductance of a transmission line is scale-invariant, 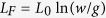, where 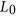 is a geometry dependent constant^6^. Thus, given a specific aspect ratio for the conductor size and separation (that is, 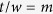 and 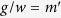 with *m* and 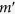 being constants), the size threshold at which the kinetic inductance exceeds the Faraday inductance is given by


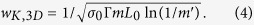


for 3D conductors and


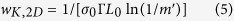


for 2D conductors. For the transmission line configurations chosen in this paper, 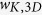 and 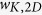 are on the order of 100 nm and 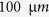 for copper and graphene, respectively. Copper’s much lower threshold is partly due to the square root dependence in equation (4) and partly due to its much larger DC conductivity (Fig. 1b). If a lower intrinsic scattering rate can be achieved, the threshold can be increased, with more mileage being gained in 2D materials.

In lines with conductors that are smaller than the thresholds given in equations (4) and (5), the onset of the LC region, 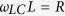 reduces to 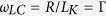. This surprisingly simple relationship suggests that the LC region and its associated constant attenuation can be found immediately above the intrinsic scattering rate. This is in contrast to transmission lines which are Faraday inductance dominated, where 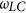 strongly depends on the geometry. Therefore, two design conditions must both be satisfied to achieve a broadband kinetic LC response: *w* < *w*_*K*_ and *ω*_*operating*_ > Γ.

**Figure 2:**
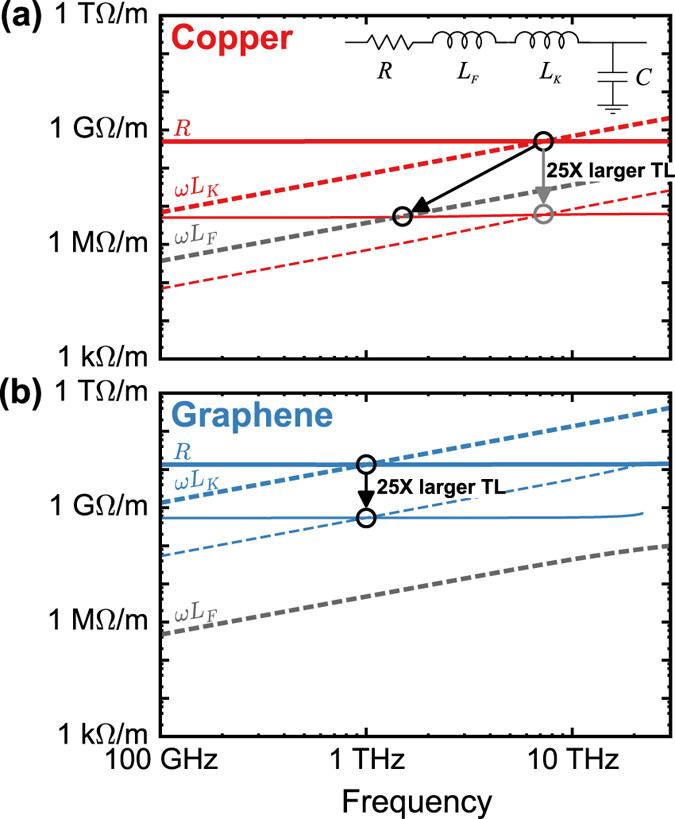
Effect of geometric scaling on the distributed circuit parameters of CPS line made of (**a**) copper and (**b**) graphene. A 25× size increase, from (w = 20 nm, g = 10 nm) to (w = 500 nm, g = 250 nm) (and additionally t = 10 nm to 250 nm for copper), reduces both R (solid curves) and 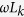 (dashed curves) by 625× for copper and 25× for graphene. 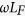 (gray curves) remains unchanged under scaling in both the copper and graphene CPS due to a fixed w/g ratio.

First-principle modeling with Maxwell’s equations, via the finite element method (see Methods section), confirms the above conditions through the inductive and resistive impedances (Fig. 2) that have been calculated directly from the TEM mode’s field distribution. We consider a 25× size scaling of a copper (Fig. 2a) and a graphene (Fig. 2b) CPS, from *w* = 20 nm to 500 nm. The conductor separation (as well as the copper conductor thickness) are equal to *w*/2, i.e. 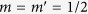 in equations (4) and (5). The resistance and the kinetic inductance of the CPS lines both decrease, by 25× in the graphene lines and by 625× in the copper lines, while the Faraday inductive impedances remain unchanged (grey curves). Both the large and small graphene CPS designs are kinetic inductance-dominated but with copper, only in the smaller of the two lines is kinetic inductance-dominated. In the graphene lines, the two electrode widths are both *below* the threshold predicted by equation (5) but the widths of the copper lines straddle the threshold given by equation (4). Thus, the *w* = 20 nm and w = 500 nm graphene CPS lines have an LC onset frequency that is exactly equal to the intrinsic scattering rate of graphene (1 THz). In the w = 500 nm copper CPS, although *R* and 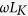 intersect at the same frequency, 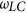
is instead determined by the intersection of *R* and 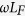, at approximately 1.5 THz.

**Figure 3:**
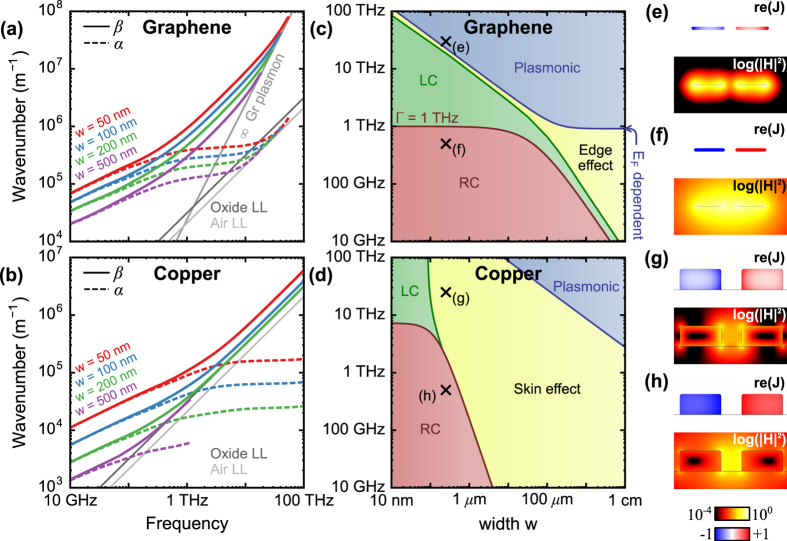
Attenuation and dispersion spectra for graphene (**a**) and copper (**b**) CPS transmission lines with various conductor widths. Performance regimes of graphene (**c**) and copper (**d**) CPS as it scales from nanoscale to macroscale. *g* and 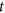 (for copper) are proportionally scaled as *g = w*/2 and *t* = *w*/2. The magnetic energy density and conduction current on the graphene and copper CPS designs are plotted at high frequencies (**e**,**g**) and low frequencies (**f**,**h**). The corresponding frequencies and widths for (**e**–**h**) are indicated by marked points in (**c**,**d**).

The above line classifications are also reflected in the spectra of 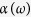 and 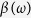 that were calculated from modal eigenvalue simulations. We consider copper and graphene CPS’s in Fig. 3a,b with several electrode widths in addition to those from Fig. 2. The LC onset corresponds to the divergence of 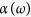 and 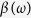 from their shared values in the RC region. All of the graphene CPS’s (Fig. 3a), regardless of width, have LC regions that start at precisely the intersection frequency of 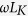 and *R* in Fig. 2(a); the copper CPS’s have an LC onset frequency that shifts to lower values for larger electrode sizes (Fig. 3b). This indicates that most of the electrode widths that we have considered result in a dominant Faraday inductance in copper. In fact, only the copper CPS with the narrowest width, *w* = 50 nm, exhibits a clearly dominant kinetic inductance while the w = 100 nm CPS lies on the threshold that was predicted by equation (4).

The high frequency boundary of the LC region in nanoscale transmission lines is determined by a different set of mechanisms than in micro and macroscale transmission lines. In copper and other metals, for frequencies below their intrinsic scattering rate, Γ the skin depth is collisional and scales as 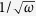. However, above Γ the skin depth converges to a fixed value (around 100 nm for copper), which is known as the collisionless or surface-wave skin depth^20^. In copper transmission lines with electrode sizes below 100 nm, no skin effect is observed (Fig. 3b), and the kinetic LC region extends into the plasmonic regime at optical frequencies.

In nanoscale graphene lines, the upper frequency limit for the LC region is *not* defined by the skin effect. At high frequencies, as 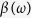 of the TEM mode approaches the graphene plasmon band (Fig. 3a), the self-capacitance introduces significant intra-electrode fields and lateral currents. This effect is similar to the transition between metal optics and plasmonics in metallic nanostructures^20^. This so-called capacitive “edge effect” increases attenuation sharply beyond approximately 30 THz.

### Transmission line performance map

For graphene (Fig. 3c) and copper (Fig. 3d), the overall landscape of TEM wave propagation and attenuation is defined by the boundaries separating the previously unrecognized kinetic-LC region (green), the conventional RC region^14^ (pink), the skin/edge effect region (yellow), and the plasmonic region^23^ (blue). All of the transmission line dimensions are proportionally scaled with respect to the electrode width, along the horizontal axes, from ten nanometers up to one centimeter. We reiterate that the specific value of Γ or 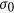, while it can cause a spectral shift of the boundaries between different regimes, does not affect the existence of these regimes or the trends and main points presented in this paper.

The lower bound of the LC region (the dark red curve), 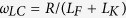, manifests itself as a size-independent horizontal line at nanoscales, given by the conductor’s scattering rate, where the kinetic inductance dominates. When the width exceeds the threshold of 100 nm for copper and 100 μm for graphene, the line becomes Faraday inductance-dominant and the LC onset is strongly width-dependent. The slope of this transition is a function of the material dimensionality with a steeper *w*^−2^ dependence in the copper CPS and a more gradual *w*^−1^ dependence in the graphene CPS. At very low frequencies in the RC region, the current distribution is highly uniform and the mode energy is concentrated predominantly in the region between the conductors (Fig. 3f,h).

The upper bound of the LC region (the dark green curve) is defined by the onset of the skin or edge effect region where the current shifts strongly towards the surface of the conductor (Fig. 3g). For the copper line, this boundary is defined as the frequency at which the skin depth is equal to half of the electrode width, 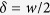. This boundary follows the same, *w*^−2^ geometric dependence of the LC region onset. In copper at frequencies above the intrinsic scattering rate, this boundary becomes a vertical asymptote as the skin depth converges to its constant collisionless value^20^. Therefore, the overall behavior of copper-based lines is characterized predominantly by the skin effect in lines with electrode widths exceeding 100 nm, and by a surprisingly large bandwidth kinetic-LC region at frequencies above the scattering rate. For graphene, the LC region transitions into the edge effect rather than the skin effect region. We define the lower boundary of the edge effect region empirically as the frequency at which the numerically computed resistance exceeds that of a spatially uniform current by 5%. This is essentially a measure of how non-uniform the current distribution becomes. Above the graphene scattering rate, the edge effect boundary has approximately a *w*^−0.5^ dependence, while below the graphene scattering rate, it closely follows the RC-LC boundary. This strongly suggests that graphene ribbons can exhibit edge-current crowding whenever their widths exceed 100 μm.

The third boundary for graphene (the dark blue curve) separates the skin/edge effect region from the plasmonic region. In the plasmonic region the transmission line becomes multi-moded since the geometry is capable of supporting individual and hybridized ribbon plasmons. For graphene, this boundary occurs when the wavelength of an infinitely-wide-graphene plasmon falls below twice the ribbon width, and runs parallel to the LC-edge-effect boundary. This implies that the observed edge effect is associated with the transition from quasi-TEM modes to ribbon plasmons where the field concentrates highly on the graphene and the current becomes non-uniform (Fig. 3e). In the macroscale graphene lines that we consider (Fig. 3d, right), this cutoff approaches a frequency nearly equivalent to the graphene intrinsic scattering rate. In general, however, this asymptotic value will be influenced by the magnitude of graphene’s conductivity through its Fermi level. In Fig. 3d, the horizontal portion of the boundary overlapping with the scattering rate is coincidental.

In copper, the cutoff for guided plasmonic modes lies at much higher frequencies for a given waveguide size, or much larger waveguide sizes at a given frequency, due to copper’s larger DC conductivity. However, at microwave frequencies, the more practical boundary is the one that separates single-mode (TEM) operation form multi-mode (TEM+TE/TM) operation. If we contrast the overall performance of graphene transmission lines to their copper counterparts, we observe that graphene lines can provide a much lower onset frequency for the kinetic LC region. Furthermore, graphene’s LC region is supported in much larger lines, up to widths of about 100 μm. For larger structures, graphene’s reduced dimensionality also results in a more gradual shift towards a Faraday inductance-dominated LC response.

High quality graphene with a sub-THz scattering rate would be extremely valuable for realizing nanoscale traveling-wave transducers^4^ and sensors, which would otherwise be in the RC region with 3D conductors. On the other hand, when the absolute lowest attenuation is desired and dispersive attenuation can be tolerated, copper (and other metal) transmission lines are more preferable. Multilayer graphene can be used to reduce attenuation^35^, but will also present a reduced kinetic inductance; multilayer graphene transmission line performance will scale similarly to 3D copper. Finally, it’s worth noting that at conductor sizes below 100 nm, copper lines will suffer from a reduced conductance due to scattering at grain boundaries and surface roughness^36^. These effects have been partially accounted for in this paper through a reduced copper DC conductivity of 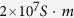. Graphene will also encounter deviations from the Drude model in sub-100 nm widths through contributions from conducting edge modes^37^,^38^ and changes in the electronic bandstructure. Although these effects are expected to shift the propagation-constant spectra, the overall propagation regimes depicted in Fig. 3 and the existence of a constant-attenuation LC region remain unchanged.

Finally, it is worth pointing out that in comparison to graphene plasmons, which are known for their exceptional wavelength reduction^21^,^25^, the TEM modes of a graphene transmission lines exhibit even greater wavelength reduction in the few-THz frequency range (Fig. 3a). The dispersion relation of a graphene plasmon^22^ suggests a low-frequency radiative cutoff (e.g. 1.5 THz for *E*_*F *_= 0.4 eV, see Fig. 3a), while the TEM mode remains guided throughout the entire spectral range. Finite-width graphene ribbons support plasmons possessing even larger *β* than those of the infinitely-wide graphene films, and therefore experience even higher cut-off frequencies. Thus, in the spectral range between 1 and 10 THz, the graphene TEM mode provides the optimal combination of wavelength-reduction, linear phase propagation, and constant attenuation.

## Conclusion

In conclusion, our analysis of graphene and copper transmission lines has revealed that the well-known TEM modes enjoy a previously unnoticed broadband constant-attenuation regime, when the lateral dimensions are reduced to the nanoscale for both 2D and 3D conductors. This so-called kinetic-LC regime is induced by a predominant kinetic inductive impedance. The threshold dimension for this regime in a 3D line is defined by the collision-less skin depth of the conductor, about 100 nm for copper. Graphene transmission lines are remarkable in that their kinetic-LC region extends to relatively large micron-scale widths. The onset frequency of the kinetic-LC region is fixed at the free-carrier scattering rate, a material property, and therefore high-quality graphene with a low scattering rate would be desirable for THz/sub-THz applications. The upper frequency cutoff, on the other hand, depends on the material dimensionality: 2D graphene lines exhibit a width-dependent upper cutoff induced by plasmonic edge effects, whereas 3D lines have no upper cutoff in the spectral range in which the Drude model is valid. Although graphene lines exhibit constant attenuation at larger dimensions, copper lines of comparable widths provide lower attenuation. However, graphene’s large kinetic inductance drastically reduces the mode wavelength by 10–40 fold, approaching 1 micron at 10 THz. This deep subwavelength operation and the inherent tunability of graphene open up new opportunities for conventional signaling and sensing applications, as well as emerging applications involving metamaterials.

## Methods

### Finite element method

COMSOL Multiphysics, a commercially available finite element solver, was used to numerically compute the modal electromagnetic field distributions. A weak formulation was developed to solve for the propagation vector as a function of frequency in the presence of graphene and other dispersive materials. In our model, graphene was implemented with a surface weak term contribution for an infinitesimally thin conductive layer, subjected to a surface conductivity boundary condition 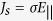, where 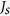 is given by the tangential magnetic field discontinuity. A fine mesh (about 20 quadratic elements per electromagnetic wavelength around the metallic electrodes) was used to resolve the spatial variation of the fields. Additionally, the mesh was regenerated as the dimensions of the transmission line were scaled from micro- to macroscale. All simulations were done in 2D with the uniform third direction (*z*) being taken into account by replacing 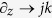 and casting the equation as an eigenvalue problem with *k* as the eigenvalue. The circuit parameters (*C*, *R*, 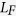, and 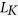) were computed by integrating the numerical electromagnetic fields to find a voltage, current, electric energy (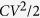), magnetic energy (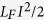), and power dissipated or stored in the conduction currents.

## Additional Information

**How to cite this article**: Mousavi, S. H. *et al.* Kinetic inductance driven nanoscale 2D and 3D THz transmission lines. *Sci. Rep.* **6**, 25303; doi: 10.1038/srep25303 (2016).
